# The Appendix: A Rare Case of an Appendiceal Collision Tumor

**DOI:** 10.7759/cureus.17050

**Published:** 2021-08-10

**Authors:** Samuel G Ruiz, Feargal Geraghty, Dalina Padron, Daniel Chacon, Gerardo Kahane

**Affiliations:** 1 General Surgery, Kendall Regional Medical Center, Miami, USA; 2 Trauma, Kendall Regional Medical Center, Miami, USA; 3 Anesthesiology, Mount Sinai Medical Center of Florida, Miami Beach, USA; 4 Medicine, Ross University, Bridgetown, BRB; 5 General Surgery, Aventura Hospital and Medical Center, Aventura, USA

**Keywords:** appendix, neoplasm, appendectomy, collision tumor, adenocarcinoma, neuroendocrine tumor

## Abstract

Appendicitis is a common cause of right lower quadrant pain. However, appropriate diagnostic evaluation and a high clinical suspicion can reveal alternative etiologies that are not so commonly encountered. In this report, we present a rare case of an appendiceal collision tumor involving two distinct neoplasms as the source of the patient's pain and describe how thoughtful clinical maneuvering led to its diagnosis and treatment.

## Introduction

Appendiceal neoplasms are a rare group of neoplasms that account for less than 1% of all gastrointestinal (GI) malignancies [1]. These occur more commonly in adults during the third decade of life and have a variable presentation. About 50% of patients present with classical symptoms of acute appendicitis and are diagnosed during surgery and histologic evaluation [2]. Notably, epithelial neoplasms and neuroendocrine tumors (NETs) are the most common appendiceal tumors; malignancies such as lymphoma, neuroectodermal tumors, nerve sheath tumors, mesenchymal tumors, Kaposi sarcoma, and metastasis are rarely encountered [3].

In addition to this uncommon entity, there also exists an even rarer presentation of appendiceal tumors known as collision tumors. It is so rare that our literature search yielded only four reported cases in the literature. Collision tumors are characterized by a bi-clonal derivation resulting from two distinct but adjacent neoplasms [4]. We present a case of an appendiceal collision tumor discovered during the evaluation of a patient presenting with acute appendicitis.

## Case presentation

**Figure 1:**
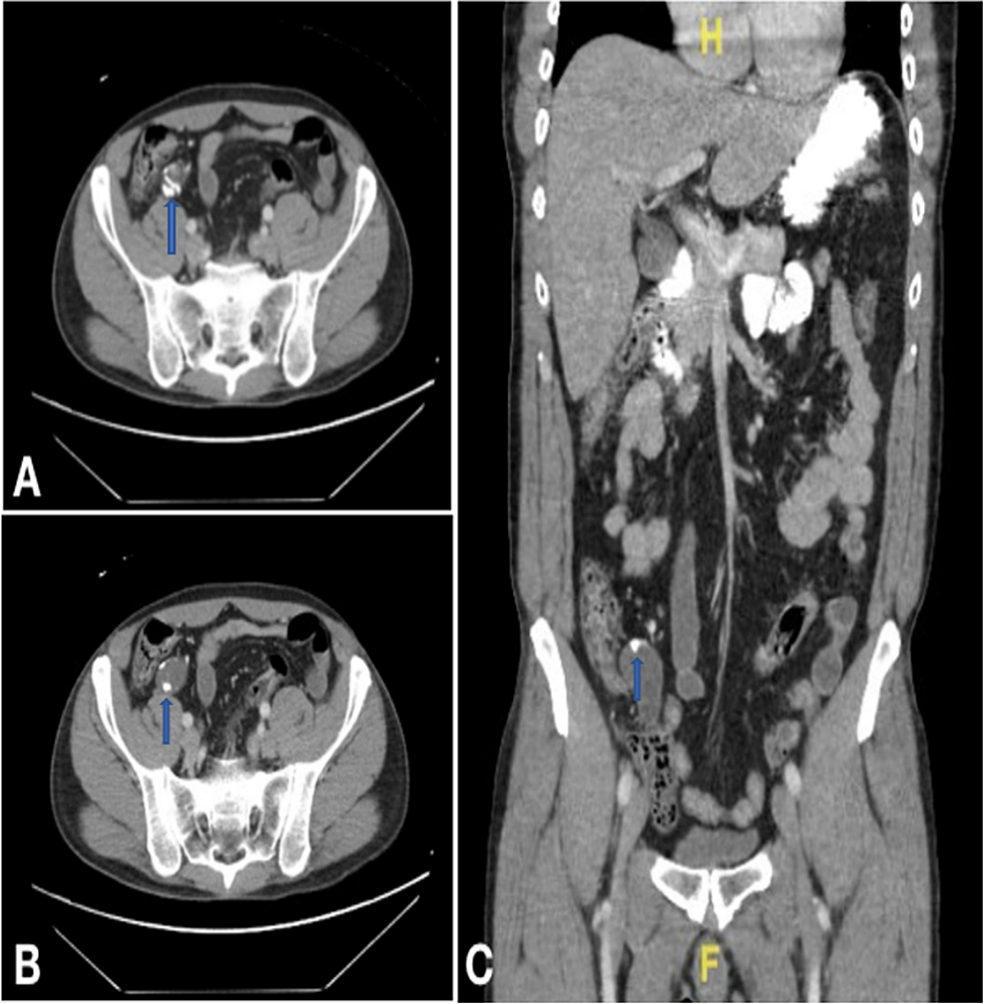
Admission CT of abdomen and pelvis A. Axial format. B. Axial format. C. Coronal format CT: computed tomography

The patient was a 54-year-old Hispanic male who presented with a one-day history of sudden onset of severe right flank and right lower quadrant abdominal pain with radiation to the right inguinal area accompanied by nausea and vomiting. The patient denied any fever or chills and no significant family history was reported. On examination, the abdomen was soft with minimal tenderness on deep palpation of the right lower quadrant without guarding or any distention. Laboratory workup revealed evidence of elevated creatinine, but leukocyte count was within normal limits. A contrast-enhanced CT of the abdomen and pelvis revealed an obstructing stone at the right ureterovesical junction (Figures 1A-1C). The appendix was visualized to be a well-circumscribed and partially calcified cystic structure measuring 3.2 x 2.5 x 4.5 cm; there was no radiographic evidence of inflammatory changes.

The patient’s clinical picture was initially attributed to ureterolithiasis and was treated accordingly. However, the cystic structure on the appendix was concerning for a mucocele. Consequentially, the decision was made to take the patient to the operating room for a diagnostic laparoscopy with a possible appendectomy. Intraoperative findings were significant for an enlarged appendix (measuring approximately 8 x 1.3 cm) with a thickened partially calcified wall, without evidence of inflammation. An enlarged and elongated cystic structure was noted in the mid-appendix. The rest of the abdominal cavity was unremarkable, without mucin or free fluid noted. A laparoscopic appendectomy was performed in the usual fashion. However, given the intraoperative findings, the decision was made to include a distal segment of the cecum in the resection. No surgical complications were encountered during the procedure.

**Figure 2:**
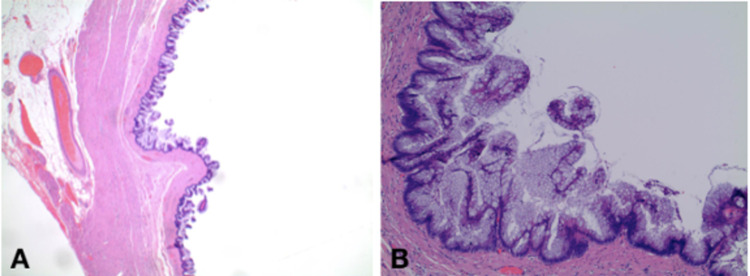
Cross-section of surgical pathology showing mucinous adenocarcinoma A. Hematoxylin and eosin stain showing mucinous adenocarcinoma. B. Hematoxylin and eosin stain showing mucinous adenocarcinoma on high power view

**Figure 3:**
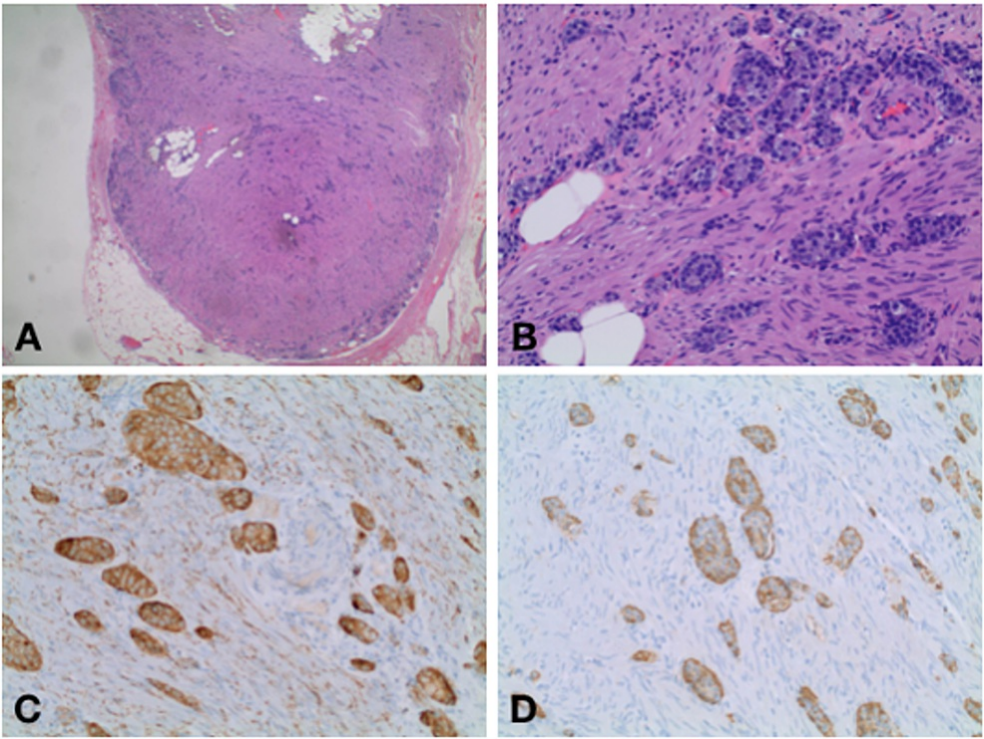
Cross-section of surgical pathology showing carcinoid tumor A. Hematoxylin and eosin stain showing carcinoid. B. Hematoxylin and eosin stain showing carcinoid on high power view. 3C. Synaptophysin stain. 3D. Chromogranin A stain

The patient's postoperative course was uneventful and final pathology was obtained three days after surgical intervention (Figures 2A, 2B; Figures 3A-3D), reporting a well-differentiated low-grade appendiceal mucinous adenocarcinoma confirmed by the muscularis propria with an estimated size of 1.2 cm, as well as a well-differentiated NET on the distal half of the appendix invading the subserosa without the involvement of the visceral peritoneum or lymphovascular invasion with an estimated size of 1.1 cm and a mitotic rate of less than 2 mitoses per 10 HPF and a KI-67 of less than 3%. The margins were uninvolved by the tumor. Surgical resection was considered appropriate, and no further treatment was required.

## Discussion

Primary appendiceal neoplasms are a rare entity, accounting for less than 1% of all GI malignancies. Clinically, the presentation may range from asymptomatic to acute appendicitis. Often, the diagnosis is made intraoperatively or during the postoperative period via histologic evaluation. Depending on which subtype of tumor we encounter, the majority of cases occur in patients during the third decade of life. Therefore, it is imperative to suspect these neoplasms, especially when it occurs in middle-aged patients. The surgical management of appendiceal tumors varies depending upon several factors, including size, location, invasion, and subtype, and can range from a simple appendectomy to a right hemicolectomy. Hence, knowing the type of appendiceal tumor is important as it guides the subsequent management. The most common appendiceal tumors are epithelial neoplasms and NETs.

Although a rare malignancy accounting for only up to 0.2% of all appendectomies and up to 6% of all appendix malignancies, epithelial adenocarcinomas are the most common type [5]. Epithelial adenocarcinomas can be classified into four subtypes: goblet cell carcinoma, mucinous adenocarcinoma, signet ring cell carcinoma, and colonic type adenocarcinoma. Carcinomas of the appendix are predominantly a well-differentiated mucinous adenocarcinoma and commonly result in pseudomyxoma peritonei without metastasis until later in the disease process. This differs from adenocarcinoma of the colon and rectum in that pseudomyxoma peritonei rarely develops and it metastasizes often [6]. Mucinous appendiceal adenocarcinomas most commonly occur in patients in the fifth and sixth decades of life. Benign tumors tend to be asymptomatic or are incidentally identified during clinical workup or histologic evaluation [3]. Malignant tumors are more commonly symptomatic, as they may present with abdominal pain in the right iliac fossa or as a palpable mass [1]. However, the most common presentation of appendiceal neoplasms is acute appendicitis. CT imaging and MRI are considered appropriate techniques in detecting mucinous neoplasms. They are characterized by mucin secretion leading to a collection of fluid recognizable on imaging studies as a mucocele, pseudomyxoma peritonei, peri-appendiceal, or localized mucinous deposits [3]. Non-mucinous epithelial neoplasms resemble colon adenocarcinomas, such that on histological and radiological examination, they are noted as a mass with the potential for regional lymph node metastasis [3]. 

It is of key importance in the treatment to consider the presence of pseudomyxoma peritonei in patients with a mucinous neoplasm. Pseudomyxoma peritonei is classically described as the presence of mucin within the peritoneal cavity; however, it is typically only described when the condition is widespread, as opposed to cases where only a small pull of mucin surrounds the appendix and in which the epithelial tumor cell is microscopically identified [7]. As described in our case, no mucin or fluid was noted in the peritoneal cavity indicating the presence of pseudomyxoma peritonei. The treatment of mucinous adenocarcinoma of the appendix entails different factors. For localized, non-ruptured, and low-grade tumors, curative measures include an appendectomy with en bloc resection of the neoplasm. If the lesion is shown to be a high-grade category of T2 or greater, a right hemicolectomy is recommended due to the increased risk of lymph node metastasis [8]. In the present case, the tumor was confined to the muscularis propria, was low-grade and non-ruptured with no spillage of content into the peritoneal cavity, and was successfully treated with a laparoscopic appendectomy only.

Neuroendocrine carcinomas, classically referred to as “appendiceal carcinoid,” are tumors that originate from the serotonin-producing enterchromaffin-like cells located in the bowel wall [8]. Appendiceal NETs are more common in patients in the fourth decade of life, with a higher incidence among women: occurring 1.7 times more than in males [9]. They most commonly present at the distal end of the appendix with a smaller percentage found more proximally. NETs are usually small in size and diameter, making them difficult to visualize during imaging studies. On imaging, they enhance avidly and may contain calcifications mimicking appendicoliths [3]. Appendiceal carcinoids can cause lumen obstruction or release vasoactive substances leading to a clinical presentation of acute appendicitis [9]. Among younger patients, NETs usually present with classic symptoms of acute appendicitis, while among the older population, they are identified incidentally during histologic evaluation. This makes their preoperative diagnosis difficult. These tumors can manifest with regional nodal involvement or metastatic disease; however, they are known to be less aggressive in comparison to other GI carcinoids [3]. Appendiceal NETs stain positive for chromogranin A and synaptophysin; classification is determined by histologic evaluation, location within the appendix, and size. The neoplasms are graded by the Ki-67 proliferation index and by microscopy, by visualizing the number of mitoses. Fewer than 2 mitoses per 10 high-power fields (HPF) and a Ki-67 index of less than 3% is classified as a low-grade tumor, whereas 2-20 mitoses per 10 HPF or a Ki-67 index of 3-20% is classified as an intermediate-grade tumor. Lastly, more than 20 mitoses per 10 HPF or a Ki-67 index of greater than 20% results in a high-grade tumor [8].

Surgery is the only curative treatment for NETs [9]. Tumor size determines the indication for surgery and is the main factor considered when predicting metastatic disease. Per International Union against Cancer/American Joint Committee on Cancer (UICC/AJCC) guidelines, tumors less than 1 cm should undergo an appendectomy only, do not require follow-ups, and show a 100% survival at five years [9]. Tumors larger than 2 cm require a partial colectomy and regional lymphadenectomy [3]. Tumors greater than 1 cm but less than 2 cm represent a group where the management is more controversial [9]. As per a consensus of guidelines from the North American Neuroendocrine Tumor Society (NANET) and European Neuroendocrine Tumor Society (ENETS), a right hemicolectomy is favored for tumors greater than 1 cm but less than 2 cm when mesoappendiceal invasion, high proliferative rate, angioinvasion, positive or unclear margins, and mixed histologic features are present [3]. In our case, the tumor size was measured to be 1.1 cm; however, the mitotic rate was less than 2 mitoses per 10 HPF and a KI-67 of less than 3%, making this tumor low-grade. The patient was successfully treated with laparoscopic appendectomy, and no further treatment was required.

In addition to the previously mentioned conditions, there also exists an even rarer presentation of appendiceal tumors, known as collision tumors. These tumors are defined as two histologically distinct neoplasms of different clonal origins coexisting within the same organ. Our literature review has revealed only a few documented cases. Singh et al. in 2011 presented a case of a collision tumor where both adenocarcinoma and carcinoid were present; however, in their case, the adenocarcinomatous component displayed omental deposit and metastasis to the regional lymph nodes at the time of presentation [10]. In our case, the appendix was the bearer of two distinct neoplasms: mucinous and neuroendocrine, both confined to the appendix.

## Conclusions

The clinical presentation in the present case was concordant with the published literature regarding the highly variable clinical presentation of this tumor, highlighting the importance of maintaining a high level of suspicion when treating middle-aged patients with atypical presentations. Following intra and postoperative diagnosis, the management of our case was considered to be appropriate. Optimal management of both histologically different tumors was taken into account independently. Both tumors were defined as low-grade, non-aggressive, and confined to the appendix, and a simple appendectomy with clear margins was all that was required to appropriately treat our patient.
